# Febrile illness and bicytopenia within hours after tick-borne encephalitis booster vaccination

**DOI:** 10.1038/s41541-019-0152-2

**Published:** 2019-12-17

**Authors:** Tim Bühler, Noemi Boos, Anne B. Leuppi-Taegtmeyer, Christoph T. Berger

**Affiliations:** 1 Department of Clinical Pharmacology & Toxicology, University and University Hospital Basel, Basel, Switzerland; 2 Regional Center of Pharmacovigilance, University Hospital Basel, Basel, Switzerland; 3 Medical Outpatient Clinic, University Hospital Basel, Basel, Switzerland; 4 Translational Immunology, Department of Biomedicine, University Basel, Basel, Switzerland; 5 Vaccination Clinic, Medical Outpatient Unit, University Hospital Basel, Basel, Switzerland

**Keywords:** Fever, Pain, Haematological diseases, Adjuvants, Inactivated vaccines

## Abstract

We report the case of a 20-year-old male complaining of sudden-onset, severe headaches, fever, chills, and generalized arthralgia. He had no symptoms of a respiratory tract infection. Blood examination revealed severe leukopenia and mild to moderate thrombocytopenia. Onset of symptoms was rapid, intense, and occurred only a few hours after routine tick-borne encephalitis (TBE) booster vaccine. The question of a relationship between booster vaccine administration and the febrile illness with bicytopenia was raised. A broad range of diagnostics excluded infections and other causes for bicytopenia. Symptoms resolved within a few days, and blood counts normalized within two weeks. Due to the close temporal relationship, a transient benign bicytopenia and febrile illness as a systemic reaction to TBE vaccination was assumed. Review of the literature and adverse event reporting systems suggest that this is a very rare reaction.

## Introduction

Different subtypes of the tick-borne encephalitis virus (TBEV) are endemic in Europe, Russia, and Asia. As the name implies, TBEV, a member of the genus Flavivirus, can cause severe meningoencephalitis. Vaccination is the most effective form of protection from all TBEV subtypes. In Europe, two different adult formulations are available: FSME-Immun® CC and Encepur® N. Both products are safe, highly immunogenic and consist of inactivated whole virus vaccines (Neudörfl and K23 strain respectively) adjuvanted with aluminum hydroxide (alum). Clinical trials showed no serious adverse events,^1^ while common, benign reactions included headache, nausea, myalgia, arthralgia, fatigue, malaise, and local reactions.^2^

## Results

**Fig. 1:**
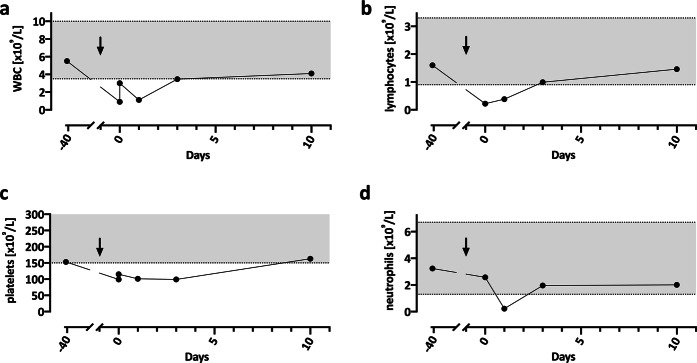
Transient, benign neutropenia in temporal relation to TBE vaccination. Total WBC (**a**), lymphocyte (**b**), platelet (**c**) and neutrophil (**d**) counts are displayed at baseline (42 days prior to vaccination) and after TBE booster vaccine (day −1). The arrow marks the administration of the TBE booster vaccine. Gray shaded areas indicate the normal range for the respective values.

We report the case of a 20-year old man who developed a transient febrile episode with chills and debilitating headaches accompanied by severe neutropenia, lymphopenia, and mild thrombocytopenia within 24 h after a tick-borne encephalitis (TBE) booster vaccine. The patient resides in Switzerland, which is an area endemic for TBE. Per the recommendations of local health authorities, he received a series of three TBE vaccinations at the age of ten. Ten years later, his general practitioner (GP) administered a TBE booster vaccination (FSME-Immun® CC, Lot. VNR1T04E). Nine hours later he started to feel feverish, experienced chills, joint pain, and severe headaches. Symptoms persisted after self-medication with a combination drug containing acetaminophen, pseudoephedrine, and dextromethorphan. The following day, he presented to his GP because of persistent symptoms. The GP recorded a fever of 39.0 °C. Complete blood count (CBC) revealed leukopenia (0.90 × 10^9^/L; range 3.50–10.00) and low platelet counts (99 × 10^9^/L; range 150–450). The patient was referred to our walk-in clinic at the medical outpatient unit. Clinical examination was unremarkable; in particular, there was no injection site reaction, lymphadenopathy, rash, signs of localized infection, or neurological abnormalities. CBC performed 20 h after vaccination revealed mild leukopenia (3.01 × 10^9^/L) as well as thrombocytopenia (115 × 10^9^/L). Forty hours after vaccination, leukocytes were again substantially lower (1.11 × 10^9^/L). In the initial blood draw in our clinic, neutrophils were normal, but with 37% bands (i.e., immature neutrophils; normal range 5.0–15.0) compared to only 52.5% segmented neutrophils (normal range 40.0–70.0). This suggests an increased neutrophil turn-over. The following day, differential blood counts showed severe neutropenia (0.22 × 10^9^/L; range 1.30–6.70) with a left-shift of 6.4%. The patient was lymphopenic on both occasions (0.22 and 0.38 × 10^9^/L, respectively; range 0.90–0.33) (Fig. 1). C-reactive protein (CRP) was only slightly elevated on the first day (11.0 mg/L; range <10), followed by a peak at 55.6 mg/L 48 h post-vaccination. Interleukin-6 (93.4 ng/L; range <7) and ferritin (581 µg/L; range 30–300) levels indicated an acute phase reaction. Tryptase was normal, and the clinical picture was not compatible with a hypersensitivity reaction.

Past medical history revealed exogenic allergic asthma and acne vulgaris, in addition to atopic dermatitis and shingles (single dermatome) at the age of 15 years. He was normal weight (71 kg). Until 4–5 weeks before the current event, the patient was taking low-dose isotretinoin (5 mg every other day) for acne treatment. Therapy was stopped due to good clinical response. Blood counts were regularly checked during the therapy and were always normal, including the most recent one taken 41 days earlier (Fig. 1).

Our initial differential diagnosis included a viral infection, a systemic reaction to vaccination, or transient bacteremia in the context of severe neutropenia. We found no clinical focus of infection. The TBE IgG level was high, with 844 VIEU/mL (positive; ≥127) and TBE IgM negative.^3,4^ However, there was no clear evidence for an immune-complex-mediated type III reaction,^5^ as complement C3 and C4 serum levels were normal, and there was no relevant local injection site reaction. We recommended supportive treatment as an outpatient with high fluid intake, acetaminophen as needed, and close clinical follow-up. White blood cell (WBC) counts normalized spontaneously within just two days, followed by platelets by day ten. Because of a persistent headache, we performed a brain MRI that excluded meningoencephalitis. All symptoms resolved spontaneously during the following days. TBE-post-vaccination titers four weeks later showed a very high TBE IgG level (>1000 VIEU/mL) and elevated TBE IgM level (2.1 S/CO). Because of the patient’s history of shingles at a young age and the current immune-dysregulated event, we completed the work-up with an immunophenotyping, which revealed normal immunoglobulin levels (IgG1–4, IgA, IgM) (Supplementary Table S1), lymphocyte subset counts (NK, CD4^+^ T cells, CD8^+^ T cells, B cells), and B cell subsets four weeks after the vaccination (Supplementary Table S2). The case was reported via the ‘Regional Center of Pharmacovigilance’ to the ‘Swiss Agency for Therapeutic Products’ (Swissmedic), the national authority of pharmacovigilance.

## Discussion

The synopsis of the available findings raises many questions, which need to be discussed in detail. Neutropenia following vaccination can be considered as a rare event. Muturi-Kioi et al. systemically reviewed clinical vaccine trials that monitored neutrophils.^6^ Neutropenia was reported after a wide range of vaccinations. These included investigational dengue and malaria vaccines, influenza vaccine,^6^ as well as an investigational live attenuated TBE vaccine.^7^ Neutropenia occurred typically in the first two weeks after vaccination, was transient (within 2–21 days) and associated with a benign outcome.^6^ Cummins et al. also reported a case of a 67-year old patient who developed severe neutropenia (0.20 × 10^9^/L) and thrombocytopenia (111 × 10^9^/L) 3 weeks after the seasonal influenza vaccination. The lab results normalized without any specific treatment. Subsequently, they studied blood count changes in 70 seniors four weeks following influenza vaccination (pre-vaccination vs. 14 and 28 days post-vaccination).^8^ They found lower WBC, but no clinically relevant cytopenia.^8^ Griffin et al. reported the case of an 83-year old woman previously treated with rituximab and steroids who developed neutropenia (nadir 0.3 × 10^9^/L) 21 days after seasonal influenza vaccination.^9^ A rituximab-associated neutropenia was, however, a likely alternative explanation in this case.^10^

Furthermore, we investigated post-marketing safety surveillance data using VigiAccess, the World Health Organization’s adverse drug reaction database. A total number of 9270 reported adverse drug reactions included only one case of bicytopenia. It is important to note, however, that the cases of the global adverse drug reaction database are spontaneous reports. Reports from the spontaneous reporting system are inhomogeneous in terms of origin, quality and completeness and are subject to reporting biases. Under-reporting of adverse drug reactions is a well-known problem. Furthermore, a lack of data concerning patient-related drug exposition prevents calculating reliable incidences of adverse reactions. Please consider that our evaluation of this case does not necessarily reflect opinions of Swissmedic, the UMC or the WHO.

Fluctuating neutropenia with a distinct left-shift and rapid resolution suggest peripheral cell depletion or redistribution rather than myelotoxicity being the primary mechanism of neutropenia. Data from clinical trials indicate that alum containing vaccines, such as the TBE vaccines, may have a higher risk for neutropenia,^6^ suggestive of an immunopathological role of innate immune activation. Alum activates the inflammasome pathway via NLRP3 signaling.^11^ Inflammasome activation can induce pyroptosis, a term describing programmed cell death via caspase-1, or induce pro-inflammatory cytokines (namely IL-1 and IL-18).^12^ Pyroptosis may occur in neutrophils.^13^ In addition to alum containing vaccines, this mechanism was also described in live attenuated vaccines.^6^ Given that our patient showed neither an immediate reaction to vaccination (i.e., within minutes) nor a strong local reaction, it remains speculative whether an innate immune activation via the inflammasome may have contributed to the neutropenia. The previous treatment with isotretinoin was an unlikely cause of the blood dyscrasias, since the drug was stopped 4–5 weeks before the detection of lymphopenia and neutropenia (half-life of isotretinoin 19 h and 4-oxo-isotretinoin, the major metabolite, 29 h).^2^ Furthermore, the patient took only a very low isotretinoin dose and had no WBC abnormalities over 2 years. Formally, we cannot exclude contamination of the vaccine as an alternative cause of the reaction. The vaccine was, however, stored appropriately, not expired, and there were no similar reactions in subjects vaccinated with the same lot. Accidental vaccine administration in a blood vessel is not to be expected at the deltoid injection site. Moreover, intravenous vaccine administration would have caused an immediate reaction. Our patient had no discomfort or symptoms during vaccine injection. Hence, we exclude intravenous injection as a cause of the vaccine reaction.

In summary, the well-documented previously normal blood counts, the close temporal relationship to the booster vaccination, and the spontaneous symptom resolution are suggestive of a systemic reaction to the vaccine. The reaction was benign, resolved without specific therapy, and had no sequela. In our opinion, spontaneous transient bacteremia or a coinciding unspecific viral infection are the most plausible differential diagnoses.

### Ethics statement

The patient gave written informed consent for the publication of his case in accordance with the Declaration of Helsinki.

### Reporting summary

Further information on research design is available in the Nature Research Reporting Summary linked to this article.

## Supplementary information

Buehler et al. Supplementary Material.pdf

Reporting Summary

## Data Availability

The raw data supporting the conclusions of this manuscript will be made available by the authors, without undue reservation, to any qualified researcher.
