# Supplementary material for: Febrile illness and bicytopenia within hours after tick-borne encephalitis booster vaccination
Source: NPJ Vaccines. 2019 Dec 17;4:52. doi: 10.1038/s41541-019-0152-2 (PMC6917779; doi:10.1038/s41541-019-0152-2)
Supplement: Supplementary file 1 — Buehler et al. Supplementary Material.pdf [file 41541_2019_152_MOESM1_ESM.pdf]

Supplementary Material

'Febrile Illness and Bicytopenia within Hours after Tick-Borne Encephalitis Booster Vaccination'

Authors: Tim Bühler, Noemi Boos, Anne B. Leuppi-Taegtmeyer, and Christoph T. Berger

Supplementary Table 1: Levels of immunoglobulins and immunoglobulin subclasses

Immunglobulin	Result (g/L)	Reference (g/L)
IgG	11.30	7.0 – 16.0
IgG1	8.36	4.9 – 11.4
IgG2	2.59	1.5 – 6.4
IgG3	0.30	0.2 – 1.1
IgG4	0.75	0.08 – 1.4
IgA	1.92	0.7 – 4.0
IgM	1.47	0.4 – 2.3

Supplementary Table 2: Flow cytometric immunophenotyping of lymphocyte subpopulations

	Absolute cells/µl	Reference	Relative (% of lymphocytes)	Reference
Lymphocyte Subsets				
T cells	1013	(742-2750)	78%	(55-86)
CD4+ T cells	536	(404-1612)	43%	(33-58)
CD8+ T cells	371	(220-1129)	30%	(13-39)
B cells	129	(80-616)	10%	(5-22)
NK-cells	145	(84-724)	11%	(5-26)
B cell subsets				
Naive B cells	86	(66-228)	66.5%	(25.1-92.4)
Transitional-B cells	4	(1-5)	2.8%	(0.3-2.9)
Marginalzone-like B cells	18	(8-172)	14.2%	(3.1-59.7)
class-switched memory B cells	14	(8-102)	10.5%	(2.4-32.6)
Plasmablasts	**6**	(1-5)	4.5%	(0.1-3.0)
CD21low B cells	2	(1-12)	1.2%	(0.5-4.7)
